# The structure and assembly mechanisms of T4-like cyanophages community in the South China Sea

**DOI:** 10.1128/spectrum.02002-23

**Published:** 2024-01-09

**Authors:** Huifang Li, Lanlan Cai, Long Wang, Yu Wang, Juntian Xu, Rui Zhang

**Affiliations:** 1 Jiangsu Institute of Marine Resources Development, Jiangsu Ocean University, Lianyungang, China; 2 State Key Laboratory of Marine Environmental Science, College of Ocean and Earth Sciences, Xiamen University, Xiamen, China; 3 Department of Ocean Science, The Hong Kong University of Science and Technology, Hong Kong, China; 4 Institute for Advanced Study, Shenzhen University, Shenzhen, China; University of Texas at San Antonio, San Antonio, Texas, USA

**Keywords:** T4-like cyanophage, community assembly, *g20*, South China Sea

## Abstract

Marine ecosystems contain an immense diversity of phages, many of which infect cyanobacteria (cyanophage) that are largely responsible for primary productivity. To characterize the genetic diversity and biogeographic distribution of the marine T4-like cyanophage community in the northern South China Sea, the T4-like cyanophage portal protein gene (*g20*) was amplified. Phylogenetic analysis revealed that marine T4-like cyanophages were highly diverse, with *g20* operational taxonomic units being affiliated with five defined clades (Clusters I–V). Cluster II had a wide geographic distribution, Cluster IV was the most abundant in the open sea, and Cluster I was dominant in coastal shelf environments. Our results showed T4-like cyanophages (based on *g20*) community was generally shaped via heterogeneous selection. Highly variable environmental factors (such as salinity and temperature) can heterogeneously select different cyanophage communities. Nevertheless, the dominant drivers of the T4-like cyanophage community based on the *g20* and *g23* (T4-like phage major capsid protein gene) were different, probably due to different coverages by the primer sets. Furthermore, the community assembly processes of T4-like cyanophages were affected by host traits (abundance and distribution), viral traits (latent period, burst size, and host range), and environmental properties (temperature and salinity).

**IMPORTANCE:**

Cyanophages are abundant and ubiquitous in the oceans, altering population structures and evolution of cyanobacteria, which account for a large portion of global carbon fixation, through host mortality, horizontal gene transfer, and the modulation of host metabolism. However, little is known about the biogeography and ecological drivers that shape the cyanophage community. Here, we use *g20* and *g23* genes to examine the biogeographic patterns and the assembly mechanisms of T4-like cyanophage community in the northern part of the South China Sea. The different coverages of primer sets might lead to the different dominant drivers of T4-like cyanophage community based on *g20* and *g23* genes. Our results showed that characteristics of viral traits (latent period, burst size, and host range) and host traits (abundance and distribution) were found to either limit or enhance the biogeographic distribution of T4-like cyanophages. Overall, both virus and host properties are critical to consider when determining rules of community assembly for viruses.

## INTRODUCTION

Viruses are the most abundant biological entities on the earth and play critical roles across diverse ecosystems by altering microbial population structure, mortality, and evolution (1–4). The global comparison of viral metagenomic data observed biogeographic differences of marine viruses (5, 6), such as the latitudinal biogeography of SAR11 phage (7–9). Cyanobacteria account for a large portion of global carbon fixation. They are widely distributed in aquatic ecosystems such as nearshore and pelagic environments, lakes, estuaries, and extreme ecosystems such as hot springs, polar regions, and deserts. Particularly, the cyanobacteria *Prochlorococcus* and *Synechococcus* contribute to up to 80% of oceanic primary productivity (10–12). Cyanophages are abundant and ubiquitous in the oceans, altering population structures and the evolution of cyanobacteria through host mortality, horizontal gene transfer, and the modulation of host metabolism (13, 14). Hence, understanding the mechanism controlling cyanophage biogeographic patterns is a central topic in ecology, particularly in cyanobacterial ecology.

There are two types of processes—deterministic and stochastic—that are primarily responsible for the spatial turnover of natural communities (15, 16). Deterministic processes refer to two types of selective forces; one (i.e., homogeneous selection) leads to more similar structures among communities due to homogeneous environmental pressures, while the other (i.e., heterogeneous selection) leads to less due to heterogeneous environmental pressures (17). Stochastic processes refer to homogenizing dispersal, dispersal limitation, and drift, which can obscure the turnover among microbial communities due to high dispersal; low dispersal; and random changes in birth, death, and reproduction, respectively (18). Increasing efforts are being made to address the relative importance of deterministic and stochastic processes in governing microbial community assembly (17, 19–24). In previous studies, both deterministic and stochastic processes influenced the bacterial and microbial eukaryotic communities in seawater and lakes (17, 25–27). Cyanobacteria exhibit global and seasonal distribution patterns that reflect environmental or dispersal limitations (28–30). Compared to the processes that drive prokaryotic and eukaryotic community assembly, the factors influencing viral community structure are less understood (31). Viruses have been observed to be significantly affected by determinism and stochasticity (32, 33). The impact of determinism was more important than that of stochasticity on T4-like viral community assembly in a transect from an estuary to an open sea environment (33), but stochasticity exerted a stronger effect in a gradient from subpolar to arctic regions (32). Researchers have observed that temperature, nutrients, and irradiance are important factors affecting the burst size and adsorption of cyanophages (34, 35), and therefore influence cyanophages community composition; however, the relative importance of deterministic versus stochastic processes underlying cyanophage community dynamics has been poorly studied.

The South China Sea (SCS) is one of the largest semi-enclosed marginal seas in the world. With a large amount of freshwater and nutrient input from the Pearl River and oceanic water intruding to the continental shelf, the northern part of the SCS (nSCS) is characterized by sharp physical and chemical gradients that produce spatial changes in microbial composition. Viral and bacterial abundances showed significant spatial distributions in the nSCS (36). Because of this, the SCS is an ideal environment for investigating how cyanophage community composition and its control mechanisms alter with distance. Given the complex and dynamic coastal environments, we first hypothesize that deterministic processes may better explain the variation of T4-like cyanophage community assembly in the nSCS. Our second hypothesis is that the dominance processes contributed to T4-like cyanophage community assembly based on different genes in the nSCS are different. To test these hypotheses, we evaluated the geographic pattern of the cyanophage community based on *g20* and *g23* genes in nSCS and provided a quantitative assessment of ecological processes governing cyanophage community assembly.

## RESULTS

**Fig 1:**
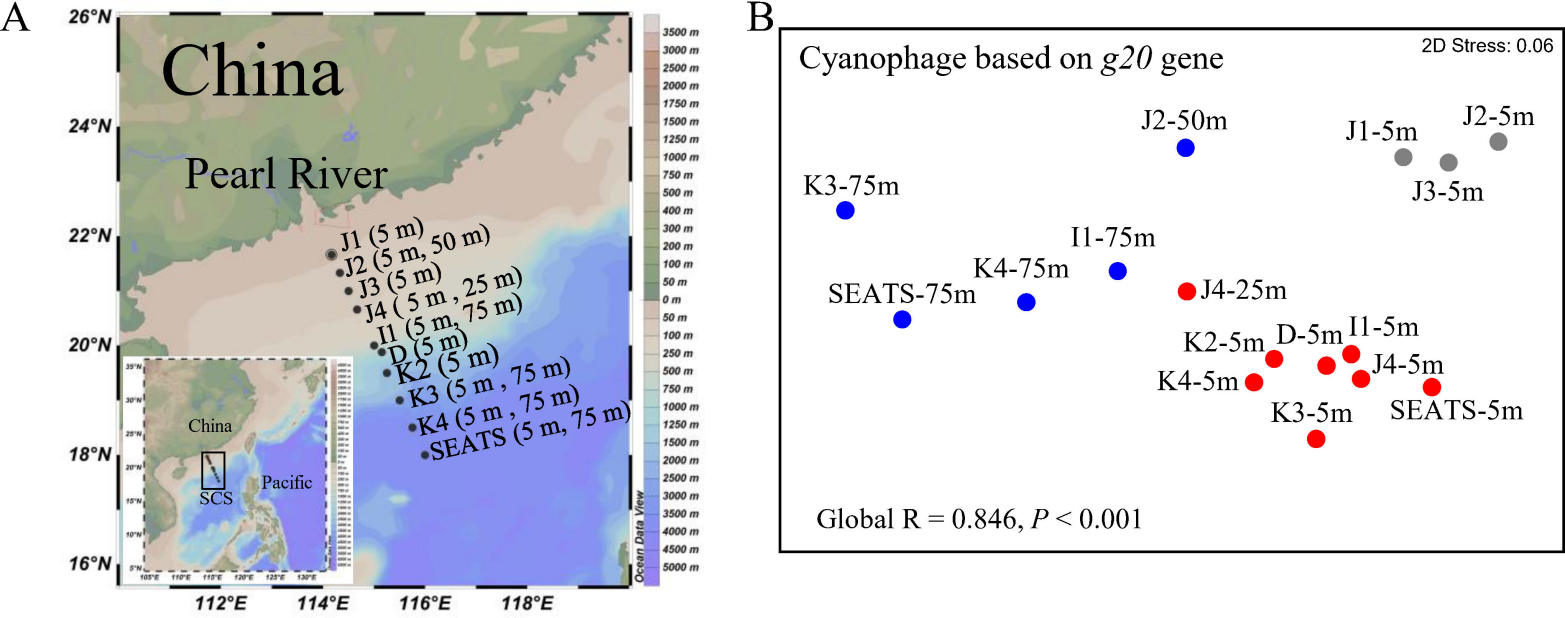
Location of the study area in the nSCS (**A**). Nonmetric multidimensional scaling (NMDS) of the T4-like cyanophage community based on all *g20* operational taxonomic units (OTUs) (**B**). Different oceanic water masses are marked in grey (CWM), red (sOWM), and blue (ssOWM), respectively.

Samples in the current study were collected from the nSCS along a gradient extending from the continental shelf to the open sea (Fig. 1A). Three water masses were defined based on temperature and salinity (33): continental water mass (CWM), a transitional region between eutrophic and oligotrophic zones (samples J1-5 m, J2-5 m, and J3-5 m), surface open sea water mass with higher salinity (sOWM; samples J4-5 m, J4-25 m, I1-5 m, D-5 m, K2-5 m, K3-5 m, K4-5 m, and SEATS-5 m), and subsurface open sea water mass (ssOWM) which had the lowest temperature (21.9–23.8°C) and highest salinity (>34.20) (samples J2-50 m, I1-75 m, K3-75 m, K4-75 m, and SEATS-75 m). Most (94.4%) of the complete genome sequence of isolated cyanomyoviruses downloaded from the NCBI database could be matched by the portal protein *g20* gene primers used in this study; because of this, it was used to investigate the genetic diversity and assembly processes of the T4-like cyanophage community.

### Diversity of T4-like cyanophage community revealed by *g20* in the nSCS

**Fig 2:**
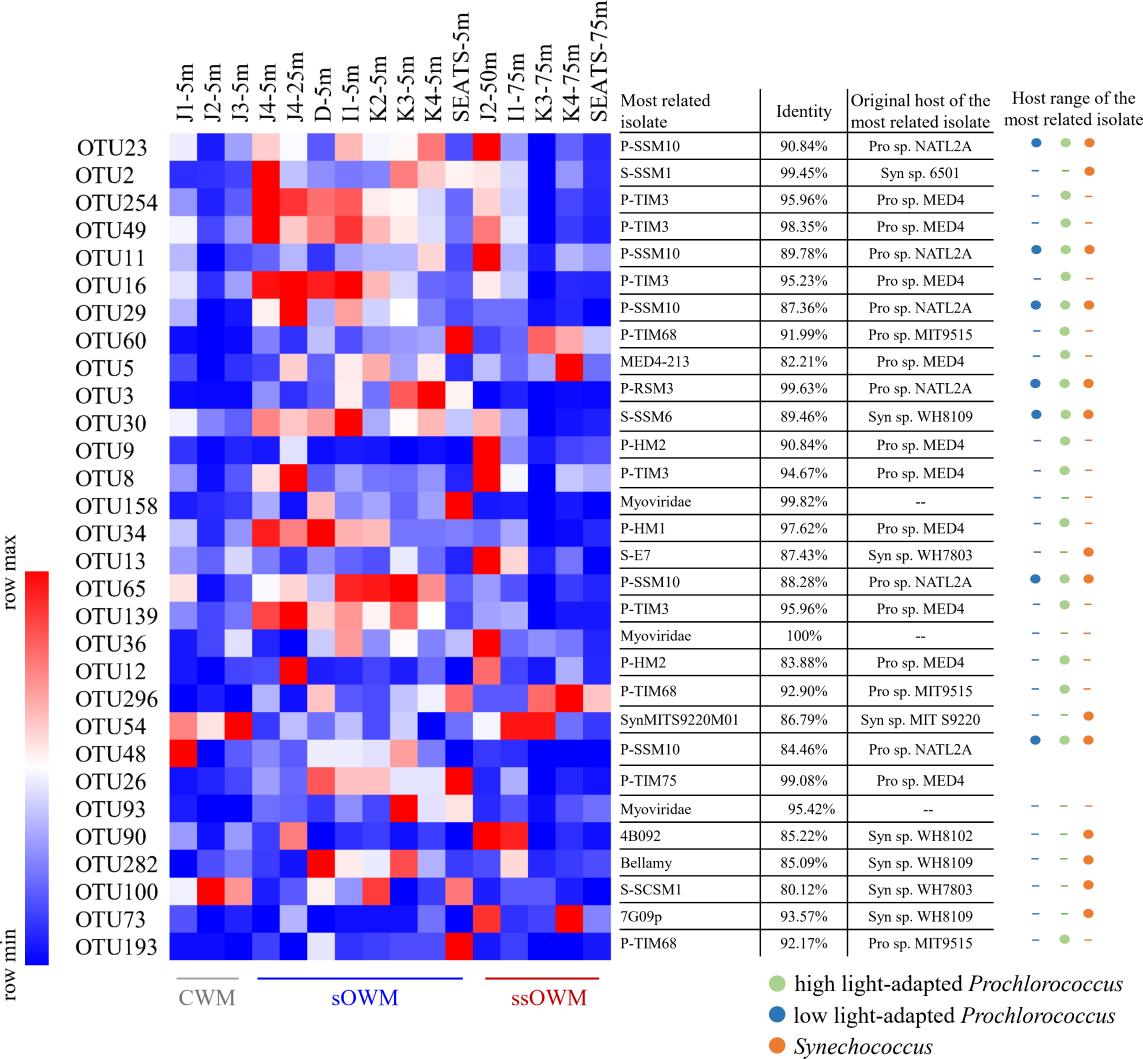
Heatmap of the *g20* OTUs that occurred in all the samples. The most related phage isolates are illustrated based on the most similar isolated cyanophage found using BlastN in the NCBI non-redundant database (*E* value < 10^−5^). The host range of the most related isolate is indicated as either high-light adapted *Prochlorococcus* (green), low-light adapted *Prochlorococcus* (blue), or *Synechococcus* (orange). The circles represent cross-infection observed within this group of hosts, whereas a dash indicates that no cross-infection has been observed.

A total of 104,330 high-quality cyanophage *g20* sequences were obtained, clustering into 789 operational taxonomic units (OTUs) based on 97% similarity. Good's coverage values of samples varied from 94% to 97%, and the rarefaction curve approached saturation (Table S1; Fig. S1). Most of the 789 OTUs were novel, with only 107 having been reported previously in different environments; these included estuary (e.g., Satilla River Estuary), coastal regions (e.g., coastal waters of Kuwait and Shantou), open sea (e.g., Sargasso Sea, Arctic Ocean, South East Pacific, and Atlantic), and lakes (e.g., Constance lake, Chilliwack Lake, and Cultus Lake) (37–39). In the nSCS, ~33% of *g20* OTUs occurred in all three water masses, while only 3.8% occurred in all samples. Among the 3.8% widely distributed OTUs, the isolated relatives of OTU 23, OTU 11, OTU 29, OTU 3, OTU 30, OTU 65, and OTU 48 (identity between 84.46% and 99.63%) could cross-infect high-and low-light adapted *Prochlorococcus* and *Synechococcus* (Fig. 2). Moreover, the related isolates of the widely distributed OTU 2 (identity of 99.45%) were those not able to cross-infect *Prochlorococcus* and *Synechococcus*, but were known to infect multiple *Synechococcus* strains, like *Synechococcus* sp. 6501 and 8012. In addition, we found a clear inverse correlation between *g20* OTU occurrence frequency and their average community contribution (Fig. S2A), and a positive relationship between average relative abundance and occurrence frequency of each *g20* OTU (Fig. S2A). For example, 51% of the abundant *g20* OTUs (average relative abundance >1% for one sample or >0.1% for all samples) appeared in over half of the samples. Furthermore, the rank abundance plot of the average contribution of per *g20* OTU was an inverted exponential curve (Fig. S2B).

**Fig 3:**
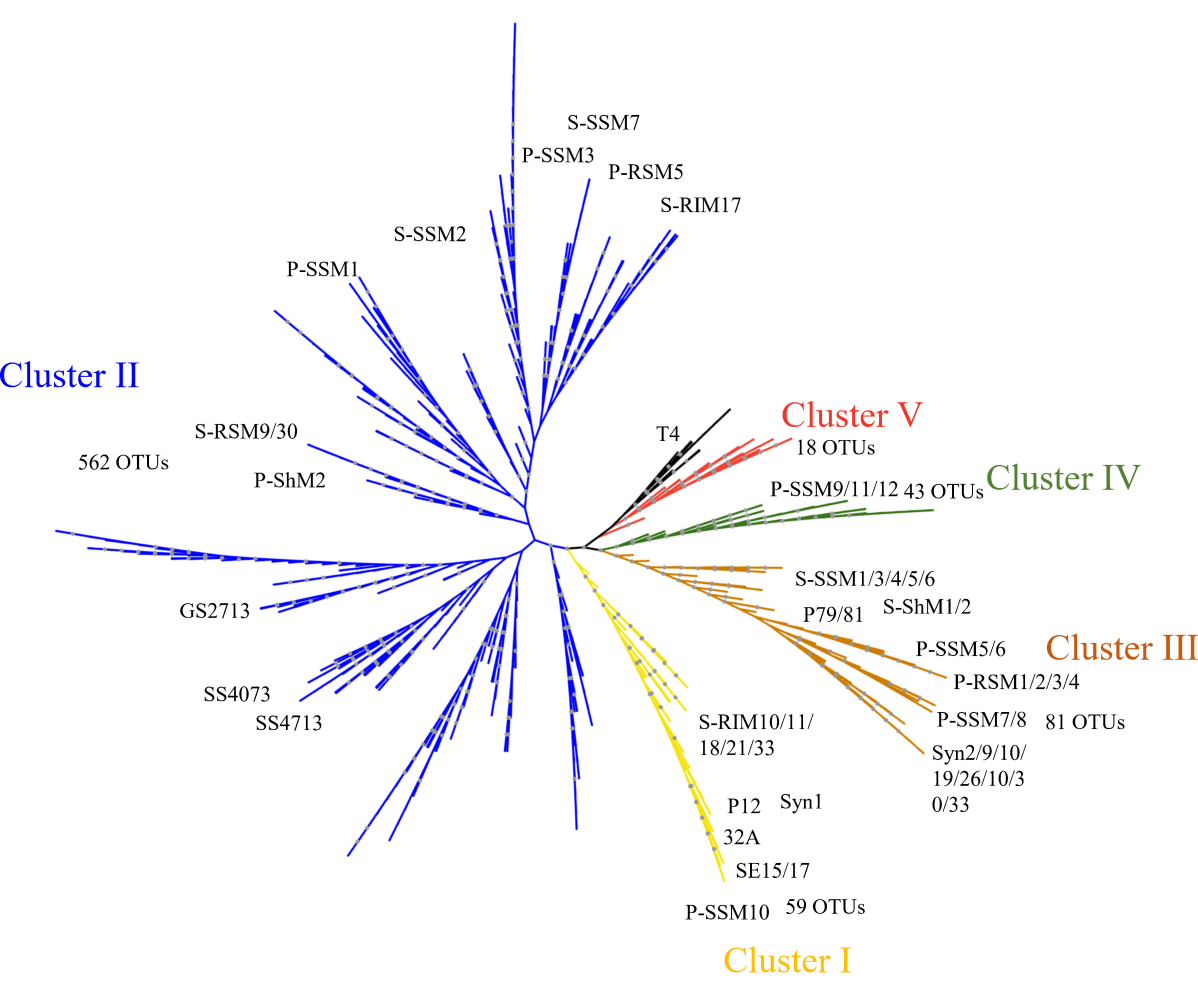
Maximum-likelihood phylogenetic analysis based on amino acid sequences of *g20* OTUs. Black dots show internal nodes with >90% bootstrap support. Different clusters are marked in yellow (Cluster I), blue (Cluster II), brown (Cluster III), green (Cluster IV), red (Cluster V), and black (Unassigned).

**Fig 4:**
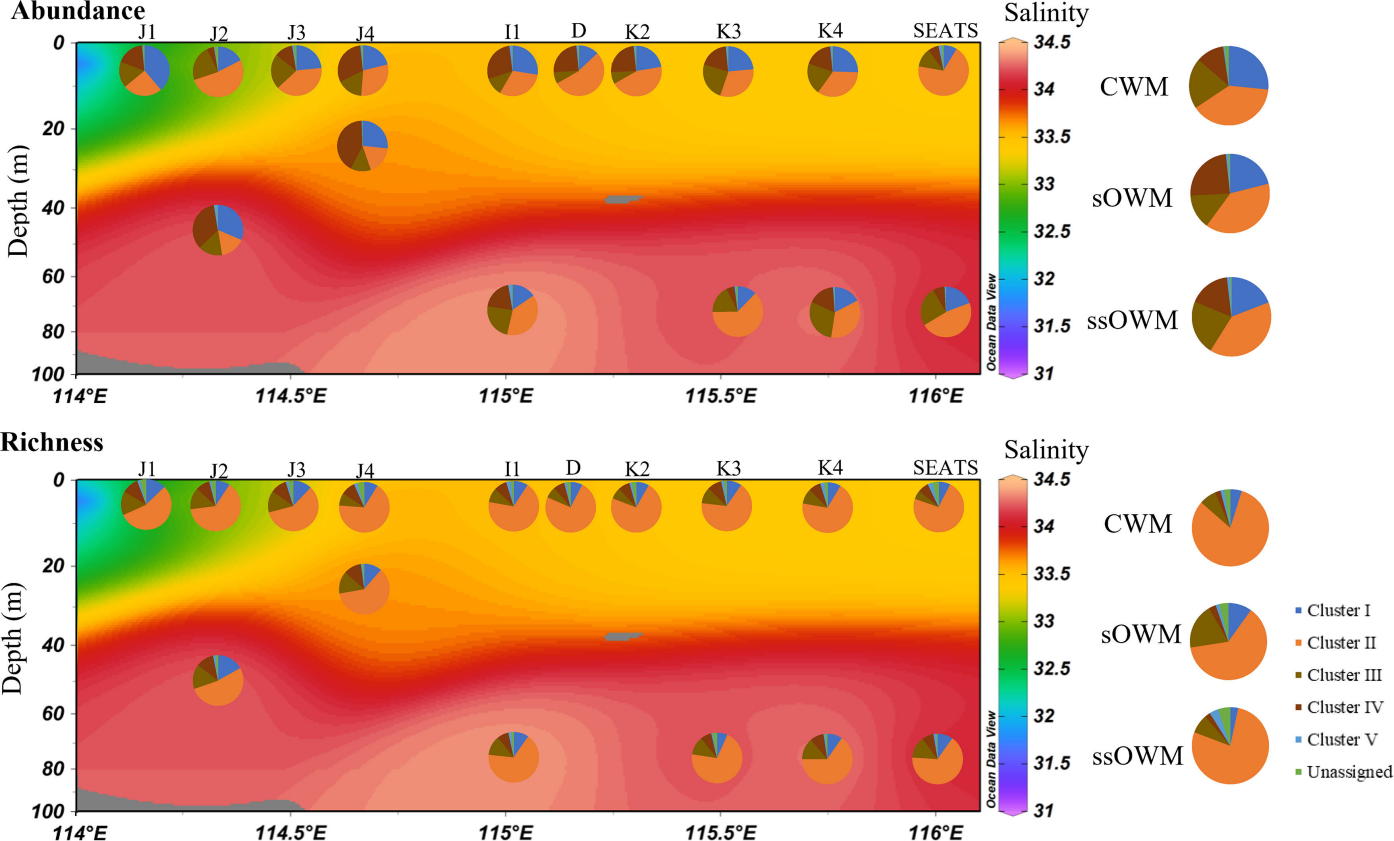
Comparison of the relative distribution of each *g20* cyanophage cluster obtained from each sample and water mass in the nSCS.

Phylogenetic analysis revealed high diversity in the *g20* OTUs obtained from the nSCS, which formed five clusters (Clusters I–V) with the environmental sequences and cyanophage isolates reported previously (Fig. 3) (11, 38–41). Clusters I–III contained cultured representatives and were defined previously by Zhong et al. (39); Cluster IV was previously defined as a new cluster by Sullivan et al. (40) and contained the isolated cyanophage P-SSM 9/11/12. Cluster V had six clades that were identified in clone libraries of natural marine communities, previously defined as environmental-sequence-only (39). The majority (89.12% of OTUs and 80.67% of sequences) of *g20* OTUs grouped with the cultured representatives within Clusters I–IV. Cluster II was the most abundant, accounting for 71.06% of OTUs and 39.30% of sequences (Fig. 4); followed by Cluster I, with 7.68% of OTUs and 22.26% of total sequences (Fig. 4). The disproportionate diversity and relative abundance of Cluster I might due to its low number of OTUs but relatively high abundance, such as OTU 23 (Fig. 2 to 4). The abundance of Cluster III (10.37% of OTUs) was slightly less than that of Cluster I, accounting for 19.09% of sequences. Moreover, the sequences of cyanophage isolates felling within Clusters I and III prefer to infect both *Prochlorococcus* and *Synechococcus*, such as P-SSM10, P-RSM2/3, and Syn 9/10/26/30 (Fig. 3). In the nSCS, 5.50% of *g20* OTUs (17.33% of sequences) fell into Cluster IV, while 2.17% of *g20* OTU (1.07% of sequences) fell within Cluster V.

### Geographic patterns of T4-like cyanophages in the nSCS

**Fig 5:**
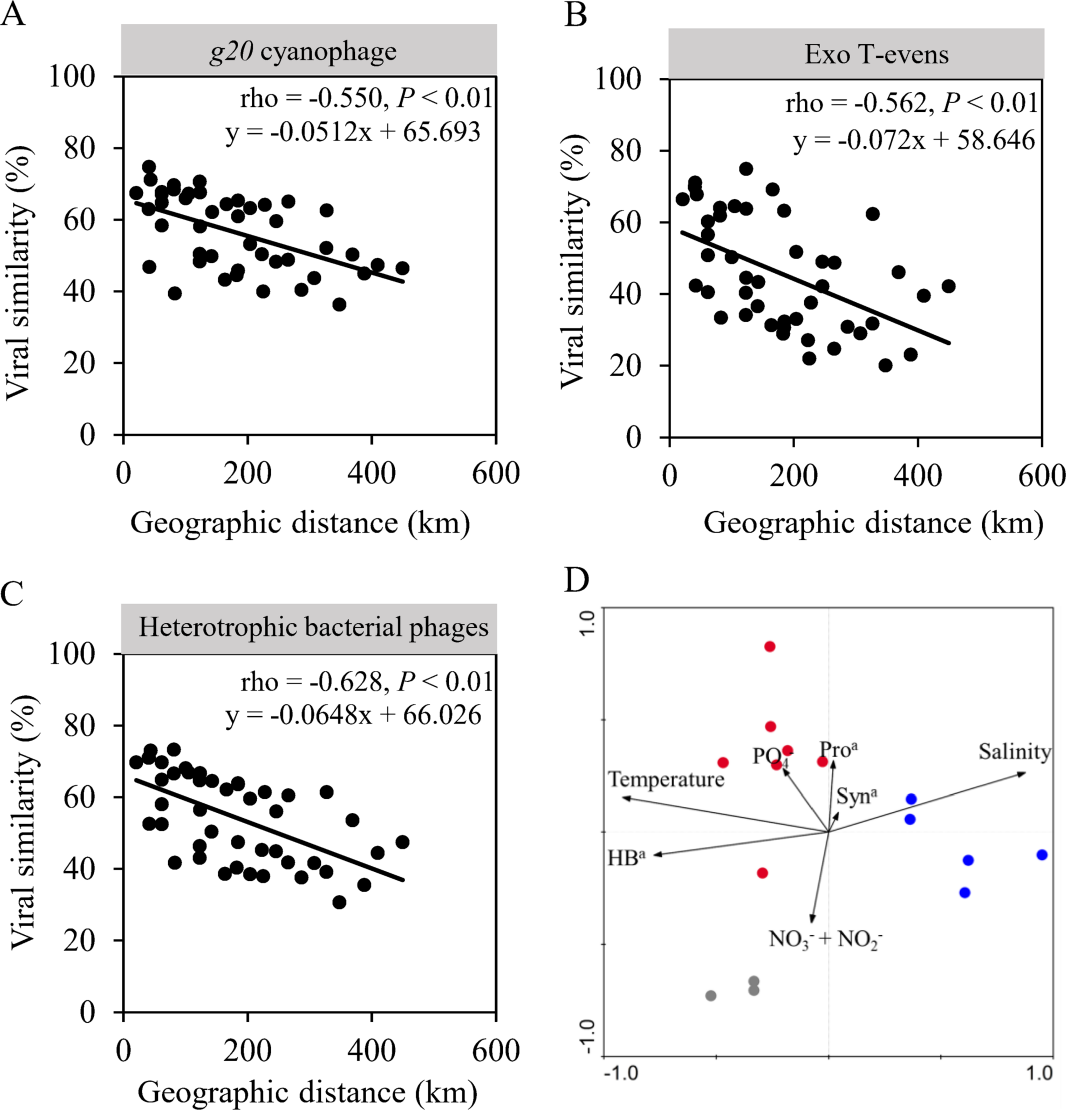
Distance-decay pattern of surface *g20* cyanophage (**A**), T4-like cyanophage (**B**), and T4-like heterotrophic bacteriophage (**C**) communities. Spearman’s correlation and significance (*P*) values are indicated. Canonical correspondence analysis (CCA) was conducted to show abiotic and abundance variables in shaping the assembly of the *g20* cyanophage community (**D**). HB^a^, Heterotrophic bacterial abundance; Pro^a^, *Prochlorococcus* abundance; Syn^a^, *Synechococcus* abundance. Samples of different oceanic water masses are marked with grey (CWM), red (sOWM), and blue (ssOWM) spots.

T4-like cyanophage community revealed by *g20* (hereafter, *g20* cyanophage) was separated into three clusters (CWM, sOWM, and ssOWM sub-communities) based on Bray–Curtis similarity (Global *R* = 0.846, *P* < 0.001) (Fig. 1B), corresponding to water mass clusters identified by temperature and salinity in nSCS (33). Similar *g20* cyanophage assemblages in geographically close samples were reflected by a clear distance-decay pattern, and significant negative correlations (*ρ* = −0.550, *P* < 0.01) occurring between pairwise Bray–Curtis community similarity and geographic distances (Fig. 5A). Moreover, a vertical stratification between the surface (sOWM) and sub-surface (ssOWM) layer was observed. Analysis of similarities (ANOSIM) showed that sample pairings of CWM and sOWM, CWM and ssOWM, and sOWM and ssOWM had significant differences in *g20* cyanophage communities (*P* < 0.05) (Table S2). Cluster II was the most abundant in all water masses, and contributed the most to the community dissimilarity between all sample pairings (Fig. S3). Sequences belonging to Cluster I were mostly recovered in CWM samples (Fig. 4), and contributed more to the community dissimilarity between CWM and sOWM and between CWM and ssOWM than sOWM and ssOWM (Fig. S3). Cluster IV was mostly recovered from the sOWM samples (Fig. 4), and had the highest contribution to the community dissimilarity between sOWM and ssOWM, followed to CWM and sOWM (Fig. S3).

### Environmental and spatial factors governing the assembly of the T4-like cyanophage community

**Fig 6:**
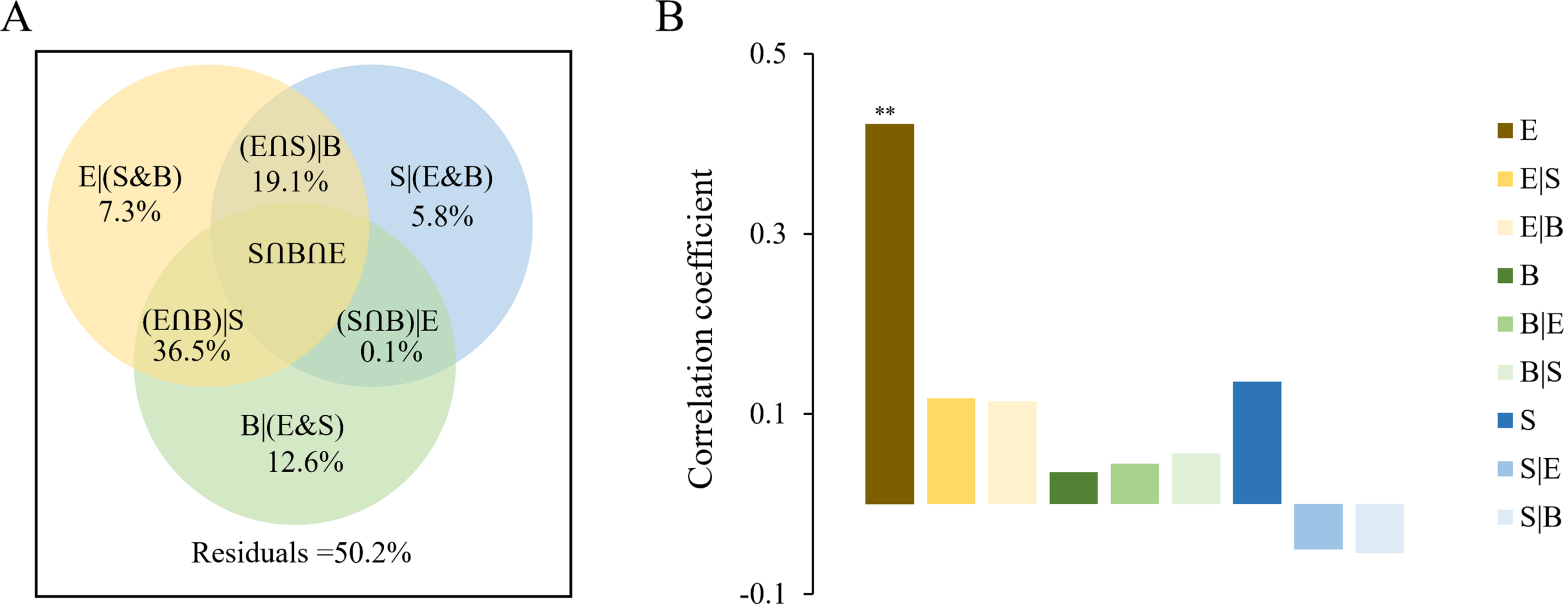
Variation in the *g20* cyanophage community explained by temperature and salinity (**E**), the cyanobacterial community (**B**), and spatial variables (**S**). (**A**) Variation partitioning analysis of the *g20* cyanophage community composition between cyanobacterial community composition, temperature and salinity, and spatial variables. The out circles represent 100% of the variation. Values < 0 not shown. (**B**) Mantel and partial Mantel tests to identify the correlations between the *g20* cyanophage community structure and cyanobacteria community composition, temperature, salinity, and spatial variables using Pearson's coefficient. “|” indicates partial mantel test. **P < 0.01.

Canonical correspondence analysis showed that three environmental variables (temperature, salinity, and heterotrophic bacterial abundance) were significantly related to the changes in the *g20* cyanophage community (*P* < 0.05) (Fig. 5D). Variation partitioning analysis (VPA) showed that the pure cyanobacterial community (B|(E&S), 12.6%) explained a higher fraction of the *g20* cyanophage variations than abiotic factors ((E|(S&B)), 7.3%), and appeared to play a greater role than pure spatial factors ((S|(E&B)), 5.8%) (Fig. 6A). Additionally, cyanobacterial community and abiotic parameters ((E∩B)|S) collectively explained a large proportion of the variation (36.5%) in *g20* cyanophage communities. Mantel tests showed that the *g20* cyanophage community was significantly correlated with abiotic variables in the environmental gradient from the continental shelf to the open sea. Both VPA and Mantel tests revealed a higher effect of environmental variables than that of spatial factors in influencing the *g20* cyanophage community assembly (Fig. 6). A large amount of unexplained variation (50.2%) was attributed to the *g20* cyanophage community. Surprisingly, there was no significant interaction between *g20* cyanophages and the cyanobacterial community (*P* > 0.05).

**Fig 7:**
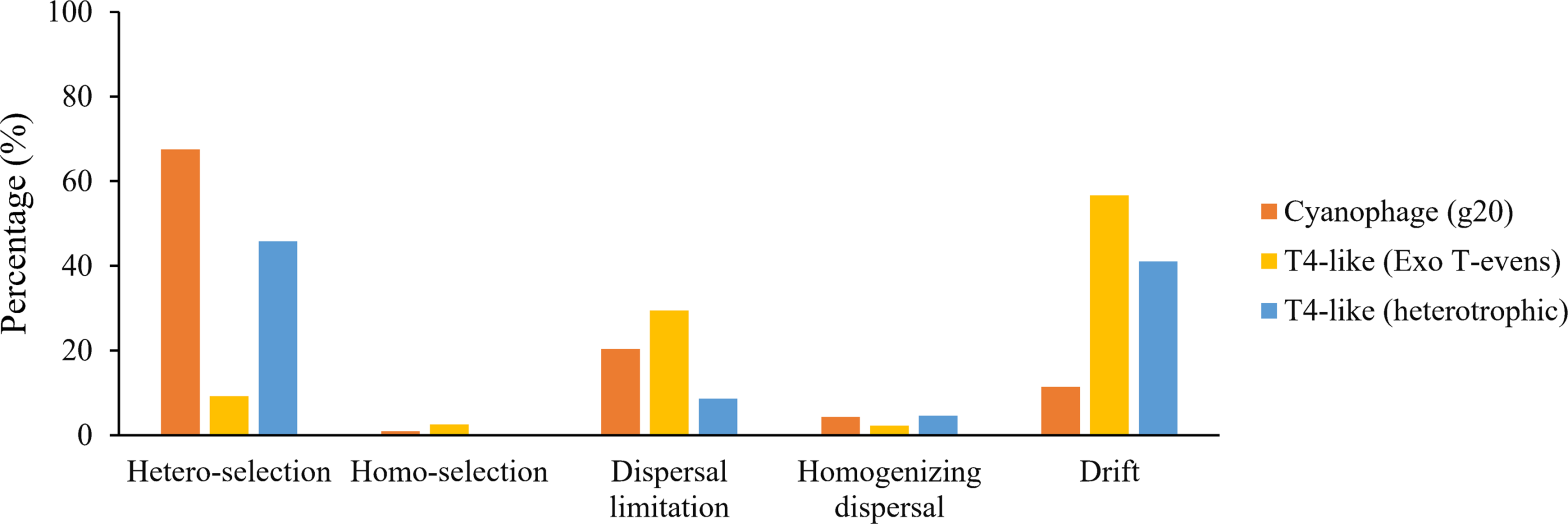
Relative importance of ecological processes in shaping the *g20* cyanophage, T4-like Exo T-evens, and T4-like heterotrophic bacteriophage communities.

Heterogeneous selection of deterministic process was more responsible for the assembly and turnover of the *g20* cyanophage community (67.50%) in the nSCS (Fig. 7); homogeneous selection, which resulted in low levels of variation in communities and was imposed by low environmental variability, contributed little. Dispersal limitation also had a higher importance in *g20* cyanophage community structuring, explaining 20.32% of the total variation (Fig. 7); this was followed by drift and homogenizing dispersal, each of which explained 11.35% and 4.22% of the community variation across all the samples, respectively (Fig. 7).

## DISCUSSION

Cyanophages control the community structure of cyanobacteria as they play a key role in regulating population density. Most isolated cyanophages belong to the T4-like group. The gene encoding portal protein, *g20*, is a signature gene applied to identify marine myovirus cyanophages, while the major capsid protein gene *g23* encompasses both T4-like cyanophages (i.e., Exo T-evens clade) and non-cyanophages (T4-like heterotrophic bacteriophage) (42, 43). Both genes have been used to investigate the diversity and spatiotemporal variations of T4-like cyanophages in a wide range of habitats such as oceans, estuaries, and freshwater environments (33, 37, 42–48).

In our previous study (33), we found that 21.5% of *g23* OTUs (25.3% of sequences) belonged to T4-like cyanophages. Among those T4-like cyanophages, only 57.4% of *g23* OTUs (66.5% of sequences) were within the isolates cluster; here, 94.4% of T4-like cyanophage isolates submitted to NCBI can be matched by the *g20* primers we used in this study. To have a better understanding of the community assembly of T4-like cyanophages, we amplified and sequenced both *g20* and *g23* genes to identify the community.

### Biogeography of T4-like cyanophage in the nSCS

There was a biogeographic distribution of *g20* cyanophage groups along the transect from the continental shelf to the open sea. We found Cluster II had a wide geographic distribution in nSCS, possibly because the hosts of cyanophage isolates in Cluster II can be widely distributed under oligotrophic and eutrophic environment (11, 39). Moreover, the cyanophage isolates S-SSM7 and S-SSM2 within Cluster II can cross-infect *Prochlorococcus* and *Synechococcus* (40). We proposed that Cluster I had a higher abundance in coastal shelf environments (26.6% of sequences) than the relatively high-salinity oligotrophic open sea (21.0% and 19.1% of sequences in sOWM and ssOWM, respectively). Additionally, the isolates that fell within Cluster I were almost all *Synechococcus* phages that were mainly isolated from Mt. Hope Bay and the Atlantic Ocean coast (Woods Hole); these areas share a similar salinity with the CWM area (11, 49). The relatively higher contribution to the dissimilarity of the sample pairings of CWM and sOWM as well as CWM and ssOWM indicated the community differences of Cluster I between the continental shelf and open sea regions (Fig. S3). The cyanophage isolate P-SSM10 and the environmental sequence references that fell within Cluster I were collected from the surface water of the Sargasso Sea, which has a similar salinity to the sOWM samples (39, 40). This might account for the relatively higher abundance of Cluster I sequences in sOWM compared to ssOWM. Cluster IV had the highest contribution (16%) to the dissimilarity between the surface and sub-surface *g20* cyanophage community (Fig. S3), which indicated the vertical difference in community structures. The cyanophage isolates that fell within Cluster IV only contained *Prochlorococcus* phage P-SSM9/11/12; its vertical distribution corresponded to the distribution of *Prochlorococcus* in the Sargasso Sea, where there was clear stratification between surface and subsurface layers in Autumn (50).

The geographic range of viruses can quickly expand or contract depending on whether their host range is broad (generalists) or narrow (specialists). Previous studies showed that the host ranges of T4-like cyanophages could vary widely, with cyanophages being strain-specific (51) or cross genera (13). We found that those abundant OTUs (30 OTUs) occurred in all the samples, and their related isolates had a relatively wide host range (Fig. 2), indicating the potential generalists; for example, OTU three with a high average abundance (1.39%) (Fig. 2) had 99.63% identity with cyanophage P-RSM3, which can broadly infect high- and low-light adapted *Prochlorococcus* strains and *Synechococcus* (40). OTU 254 (average abundance of 3.36%) had 95.96% identity with cyanophage P-TIM3, and was widely distributed in the nSCS; this cyanophage was originally isolated from *Prochlorococcus marinus* MED4 but can also infect *P. marinus* MIT9215 and MIT9312. A wide host range may contribute to the successful establishment of the virus, which might decrease the influence of dispersal limitation that will benefit their wide distribution.

The abundance of permissive hosts also affects the biogeography of their viruses. *Synechococcus* can colonize a broad range of ecological niches, extending from estuaries to the oligotrophic waters of the ocean (52–54). Consistently, OTU 2, which has a relatively high identity (99.45%) with a *Synechococcus* phage, showed a wide distribution in the three water masses. In summary, these results suggested that a virus infecting a widespread and abundant host may have fewer dispersal limitations than a virus infecting a less abundant organism occupying a narrow niche (55). Moreover, the presence of host-derived auxiliary metabolic genes in the viral genome can increase viral genetic potential, which can lead to greater viral production by increasing burst size or reducing latent period and can thereby expand dispersal.

### Heterogeneous selection dominance in shaping T4-like cyanophage community

Using the null modeling approach of Stegen et al. (56), we observed that heterogeneous selection was the most important ecological process shaping the T4-like cyanophage community in the nSCS. This scenario is somewhat expected as the nSCS is characterized by marked environmental heterogeneities; highly variable environmental factors (such as salinity and temperature) can heterogeneously select different cyanophage communities (Fig. 4D), as they play important roles in the growth and survival of viruses (57–59). Salinity changes might directly affect or alter the osmoregulation and metabolism of the microbial cells (60–62), which would affect the adsorption of viruses to their host cells (59). Viral capsids have differing permeabilities to salt ions, which can lead to inactivation or destruction when exposed to rapid changes in ionic strength (63). Furthermore, temperature affects the survival rate of free cyanophages, directly impacting their potential virulence (34). The infection rate of cyanophage is largely dependent upon the contact rate (64) and the resistance of the host cell to the infection (65). Several studies have shown that an increase in temperature led to a decrease in water viscosity, which induced a 10.7% increase in cyanophage–host contact rate (66, 67). Cheng et al. found that cyanophages in warmer waters had an over 50% increase in the efficiency of plaquing, which directly relates to infectivity (66). OTU 23 showed a higher abundance in sOWM samples than ssOWM and CWM samples (Fig. 2). The most related isolate in this OTU, cyanophage P-SSM10, was collected from the Sargasso Sea (salinity of 33 and temperature of 22–29.99°C), which has similar temperature and salinity to the surface open sea samples of the nSCS. VPA results also clearly revealed that the contribution of environmental variables to the variation in the *g20* cyanophage community was larger than that of spatial variables. These findings were consistent with the study of the T4-like viral community in the transect of the nSCS, indicating that environmental variables explained most of the community variance (33).

For the viral community, host filtering is another important selection factor due to specific virus–host interactions such as infection, co-habitation, and mutualism (24, 33, 46, 55, 68–73). The factors largely explaining cyanophage community variations were shared by both abiotic environmental factors and the cyanobacterial community (Fig. 5A). This may be because the environmental factors affecting the cyanophage community are closely tied with the changes of the cyanobacterial community, explaining why the environmental factors and cyanobacterial community together made up most of the factors influencing cyanophage community variation. There was no significant interaction between the *g20* cyanophage and cyanobacterial communities (*P* > 0.05) in this study, which might result from the lower sensitivity of 16S rDNA in amplifying the cyanobacterial community sequences in comparison to the internal transcribed spacer (ITS) fragment. Cyanophages can have broad or narrow host ranges, and differences in the host range may also be an important factor resulting in the absence of significant differences between cyanobacteria and cyanophage communities.

### Contrasting T4-like cyanophage community assembly based on *g20* and *g23* genes

Using two specific marker genes to analyze the viral community composition is conducive to a more comprehensive understanding of T4-like cyanophage community assembly. The major capsid gene, *g23,* was used to distinguish Exo T-evens type phage (T4-like cyanophage) and T4-like heterotrophic bacteriophages based on phylogenetic analysis. In the T4-like viral community, 223 *g23* OTUs (12,650 sequences) of T4-like cyanophages (Exo T-evens) and 505 g23 OTUs (26,525 sequences) of heterotrophic bacteriophages were selected based on the phylogenetic tree of *g23* amino acid sequences (Fig. S4). There was a significant separation of T4-like Exo T-evens (Global *R* = 0.772, *P* < 0.001) and heterotrophic bacteriophages (Global *R* = 0.922, *P* < 0.001) communities among the samples, with community distribution corresponding to the *g20* cyanophage community (Fig. S5). The null model showed that drift was the predominant process causing variation, accounting for 56.68% of the T4-like Exo T-evens community variation across all the samples; this was followed by dispersal limitation (29.44%). The *g20* gene explored a much greater diversity of T4-like cyanomyoviruses than the *g23* gene because the clade formed by cultured cyanophages showed almost no environmental sequences based on the phylogenetic analysis of *g23* genes. This indicated that the different T4-like cyanophage coverage of the *g20* and *g23* primers might account for the differences in the major assembly processes between the *g20* cyanophage and T4-like Exo T-evens communities.

Like the *g20* cyanophages, heterogeneous selection of deterministic process was the most important factor influencing the assembly and turnover of the T4-like heterotrophic bacteriophage community (45.83%) (Fig. 7). Dispersal limitation was more responsible for the assembly of the *g20* cyanophage community than the T4-like heterotrophic bacteriophage community. Dispersal limitation, that is, whether microorganisms show restriction in the movement and/or establishment at a new location, leads to more dissimilar structures among communities (16). Many factors could affect the successful establishment, such as environmental filtering, biotic interactions, and priority (16, 18). T4-like heterotrophic bacteriophages have been isolated from a variety of hosts, including typical heterotrophic bacteria such as *Aeromonas*, *Vibrio*, *Methylophilales,* and SAR11; in contrast, *Prochlorococcus* and *Synechococcus* are the major hosts of cyanophages and are generally distributed in the open sea and coastal sites, respectively (Fig. S6) (74). Limitations in host diversity and distribution might contribute to the larger impact of dispersal limitation on the *g20* cyanophage community assembly. Drift refers to stochastic changes in species identity and the relative abundance of different species within a community over time due to the inherently random process of birth, death, and reproduction (18). Our study revealed that drift influenced the T4-like heterotrophic bacteriophages community more than the *g20* cyanophage community (Fig. 7). Burst size, the number of viral particles released by the lysis of a cell, is another important factor affecting viral distribution (55); a short latent period and large burst size are essential features for the high proliferation of virus populations. For instance, the lytic SAR116 virus HMO-2011, which had a high burst size (500 PFU/cell), were abundant and widespread in the euphotic zone in coastal, transitional, and open regions (75). Compared to phages of heterotrophic bacteria, cyanophages have relatively long latent periods (several hours), and their burst sizes range from tens to hundreds of particles per cell (76). In contrast, T4-like heterotrophic bacteriophages have latent periods ranging from 10 min (T4-like *Vibrio* phage ϕVP-1) to 30 min (*Aeromonas* phage phiAS4) and larger burst sizes (395.8 PFU/cell for *Aeromonas* phage phiAS4) (77, 78). The large burst size and short latent period of T4-like heterotrophic bacteriophages could contribute to rapid replication with high growth and loss rates, which may be one of the most important reasons that drift has a higher impact on its community assembly than *g20* cyanophage.

### Conclusion

In this study, we determined the biogeographic distribution pattern of the T4-like cyanophage community in the nSCS. Phylogenetic analysis indicated that most of the *g20* OTUs fell within Clusters I–V. Cluster II had a wide geographic distribution. Cluster I mainly presented in coastal shelf environments, while Cluster IV was recovered most from sOWM samples and vertically distributed in surface and sub-surface layers in the nSCS. Our study highlighted the importance of examining ecological processes to understand the T4-like cyanophage community and contrasted the difference between T4-like cyanophage and T4-like heterotrophic bacteriophage community assemblies. We provided evidence that heterogeneous selection had a greater role in shaping *g20* cyanophage and T4-like heterotrophic bacteriophage community patterns than the stochastic process, and that drift was the predominant process impacting the T4-like Exo T-evens community. Furthermore, characteristics of viral traits (latent period, burst size, and host range) and host traits (abundance and distribution) were found to either limit or enhance the biogeographic distribution of T4-like cyanophages. Overall, understanding host distribution, viral host ranges, and other life history properties is critical to studies in viral biogeography.

## MATERIALS AND METHODS

### Samples collection and environmental parameters

Water samples were collected from the surface (5 and 25 m) and subsurface layers (50 and 75 m) at ten stations in the nSCS in September 2014 (Fig. 1A). Sampling stations exhibited high degrees of heterogeneity with horizontal (continental shelf and open sea) and vertical (surface and subsurface) variations (Fig. 1A). Following protocols described previously, after being pre-filtered with a 20 µm sieve, the bacteria and virus-sized particles in the filtrate were concentrated by tangential-flow filtration with a 0.22 µm filter (Millipore Corp., Jaffery, New Hampshire, USA) and a 30 kDa cartridge (Millipore Corp.), respectively. Details on the sampling sites and the sampling process have been published previously (33).

Physical parameters (temperature, salinity, and depth) and nutrients (NO_3_^−^ + NO_2_^−^, SiO_3_^2−^, and PO_4_^3−^) were measured using conductivity–temperature–depth oceanic profilers (SBE9/11 plus; Sea-Bird, Bellevue, WA, USA) and a colorimetric method, respectively. The *Pamk* function of R package *fpc* was used to cluster water masses according to temperature and salinity. Samples for the determination of microbial abundances (autotrophic picoeukaryotes, *Synechococcus*, *Prochlorococcus,* and heterotrophic bacteria) were collected and fixed using glutaraldehyde (0.5% vol/vol final concentration) at 4°C for 15 min in the dark, then were frozen in liquid nitrogen until further analysis by a flow cytometer (33, 79, 80).

### DNA extraction, PCR, sequencing, and sequence processing

Total DNA was extracted from the viral concentrate using the phenol/chloroform/isoamylol method (33). The *g20* gene from cyanophages was amplified using primer pair CPS1.1 (GTAGWATWTTYTAYATTGAYGTWGG) and CPS8.1 (ARTAYTTDCCDAYRWAWGGWTC) following a previously reported protocol (81). The PCR products were sequenced using the Pacific BioSciences platform. Circular consensus (CCS) reads were generated using ccs software v.3.0.0 (https://github.com/pacificbiosciences/unanimity/) with—minPasses 3 (82). The amplicons from different samples were then separated by barcode. OTUs were clustered in UPARSE at 97% similarity (83). OTU representative reads were translated to amino acid sequences, and sequences with termination codons were removed. Blastp was used to examine close relatives of each OTU against the NCBI non-redundant protein database with an *E* value <10^−5^. Singletons (OTUs that occurred only once) were removed, and samples were subsampled to 2,762 (minimum sample size) from the OTU table for downstream analyses. The *g20* amino acid sequences were aligned using mafft (—localpair —maxiterate 1000) (84) and then adjusted with trimAl (-automated1) (85). Maximum likelihood phylogenetic trees were built using IQ-Tree v1.6.6 with an LG + F + G4 model and an ultrafast bootstrap with 1,000 iterations (86). A phylogenetic tree for all *g23* genes was also constructed using the maximum likelihood method with IQ-Tree v1.6.6 and an ultrafast bootstrap with 1,000 iterations to pick the T4-like Exo T-evens (cyanophage) and heterotrophic bacteriophages (mainly environmental sequences from the Marine Group, Estuary Group, Lake Group, and Paddy Group) in the T4-like viral community.

Total DNA was extracted from the bacterial concentrate as previously described (87), and the conserved V3-V4 region of the bacterial 16S rRNA gene was amplified using primers 338F (5′-ACTCCTACGGGAGGCAGCAG-3′) and 806R (5′-GGACTACHVGGGTWTCTAAT-3′) (88, 89). The resulting amplicons were sequenced using the Illumina MiSeq PE300 platform. Sequenced paired-end reads were assembled with FLASH (90). Raw data were processed and analyzed using Trimmomatic and MOTHUR v.1.41.1 to remove primers and low-quality reads (91). Sequences were clustered into OTUs based on 97% sequence similarity. Those with a total community abundance >0.001% were selected for further analysis. The number of sequences from each sample was subsampled to the same amount (30,000). Classification of representative sequences in each OTU was performed using MOTHUR v.1.41.1 with SILVA (v132) reference sequences and taxonomic outlines to select cyanobacteria.

### Biogeography of cyanophages in the nSCS

Rarefaction curves based on the identified *g20* OTUs were estimated by PAST (v.3.18). The rarefied sequences were then used to determine Good's coverage and alpha diversity indices in QIIME (v.1.8.1). Nonmetric multidimensional scaling (NMDS) analysis based on the Bray–Curtis similarity of *g20* and *g23* OTUs was performed to determine the difference between the samples. ANOSIM was used to test the significance of differences among *g20* cyanophage, T4-like Exo T-evens, and T4-like heterotrophic bacteriophage communities. Similarity percentage (SIMPER) analysis was used to determine the contribution of *g20* OTUs to the observed dissimilarity between different oceanic water masses. NMDS, ANOSIM, and SIMPER analyses were performed in PRIMER 6.0 (92).

Variables with a high variance inflation factor (>10) were eliminated to avoid collinearity among factors using R package *vegan*. The relationships between *g20* cyanophage communities and environmental factors were studied using CCA, because the longest gradient length of the detrended correspondence analysis on the *g20* cyanophage community data sets was >4. Pairwise geographic distances between samples were calculated based on the coordinates of the sampling stations using a spheroidal model of Earth (32). The Euclidean distance matrices of abiotic and abundance variables, as well as the Bray–Curtis similarity matrices of the *g20* cyanophage community, were transformed using algorithms of log_(*x*+1)_ in PRIMER 6.0. Spearman's rank correlations among Bray–Curtis similarity, Euclidean distance, and geographic distances were calculated.

### Null model, VPA, and partial Mantel test

Ecological null modeling tools β-nearest taxon index (βNTI) and the Raup–Crick metric (Bray–Curtis) (RC_BC_) were applied to investigate the contribution of different ecological processes to community assembly (56, 93). β-mean nearest taxon distance (βMNTD) was used to quantify phylogenetic turnover between communities. βNTI, which is a standardized estimate of βMNTD, was calculated as the number of SDs of the observed βMNTD from the null distribution of βMNTD. |βNTI| > 2 represent the deterministic process. Values <−2 or >+2 indicated homogeneous (significantly less than expected phylogenetic turnover) or heterogeneous (significantly more than expected phylogenetic turnover) selection, respectively. βNTI values falling within the range of −2 to 2 (do not significantly deviate from the null βMNTD expectation) indicated the observed compositional difference was due to stochastic processes. RC_BC_ was calculated to distinguish these three processes, with RC_BC_ < −0.95, |RC_BC_| < 0.95, and RC_BC_ > 0.95, being interpreted as homogenizing dispersal, drift, and dispersal limitation, respectively.

VPA was used to assess the relative role of environmental and spatial factors as well as their combined effect. Temperature and salinity variables that significantly (*P* < 0.05) affected the community structure in CCA were used in VPA. A set of spatial variables were generated using the principal coordinates of neighbor matrices analysis. The pure effect of the bacterial community (B|(E&S)) (i.e., the exclusive cyanobacteria community excluding abiotic factors and spatial variables), abiotic environmental variables (temperature and salinity) (E|(S&B)), and spatial variables (S|(E&B)) were tested for significance using analysis of variance test. Mantel and partial Mantel tests were conducted to verify the significance of the results obtained from VPA. All these analyses were performed using the R package *vegan*. We have submitted the code in GitHub platform (https://github.com/812huifang/Microbiology_Spectrum.git).

## Data Availability

The raw reads of *g20* gene amplicons have been deposited in the NCBI SRA database (accession number SRR19257205−SRR19257220). The data of *g23* gene sequences for T4-like viruses and 16S rRNA genes were downloaded from the NCBI database (SRR11786658−SRR11786673 and SRR11794484−SRR11794499), which have been described in our previous study (33).

## SUPPLEMENTAL MATERIAL

Fig S1-S6, Tables S1 and S2 spectrum.02002-23-s0001.docx Supplemental figures and tables.

ASM does not own the copyrights to Supplemental Material that may be linked to, or accessed through, an article. The authors have granted ASM a non-exclusive, world-wide license to publish the Supplemental Material files. Please contact the corresponding author directly for reuse.
