# Supplementary material for: The structure and assembly mechanisms of T4-like cyanophages community in the South China Sea
Source: Microbiol Spectr. 2024 Jan 9;12(2):e02002-23. doi: 10.1128/spectrum.02002-23 (PMC10846272; doi:10.1128/spectrum.02002-23)
Supplement: Fig S1-S6, Tables S1 and S2 — Supplemental figures and tables. [file spectrum.02002-23-s0001.docx]

Supplementary Information for

The structure and assembly mechanisms of T4-like cyanophages community in the South China Sea

Huifang Li^12^, Lanlan Cai^3^, Long Wang^2^, Yu Wang^2^, Juntian Xu^1^, Rui Zhang^24^*

^1^Jiangsu Institute of Marine Resources Development, Jiangsu Ocean University, Lianyungang, China

^2^State Key Laboratory of Marine Environmental Science, College of Ocean and Earth Sciences, Xiamen University, Xiamen, China

^3^Department of Ocean Science, The Hong Kong University of Science and Technology, Hong Kong, China

^4^Institute for Advanced Study, Shenzhen University, Shenzhen, China

*Corresponding author

E-mail: ruizhang@szu.edu.cn

This file includes:

Fig. S1-S6

Tables S1-S2

**
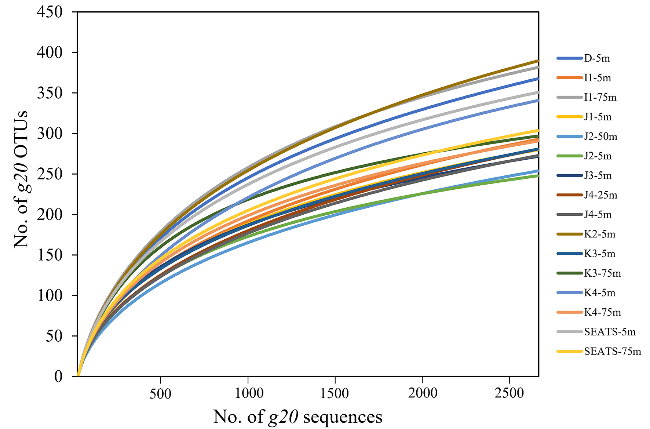
**

**Fig. S1** Rarefaction curve of similarity-based *g20* OTUs at 97% similarity.


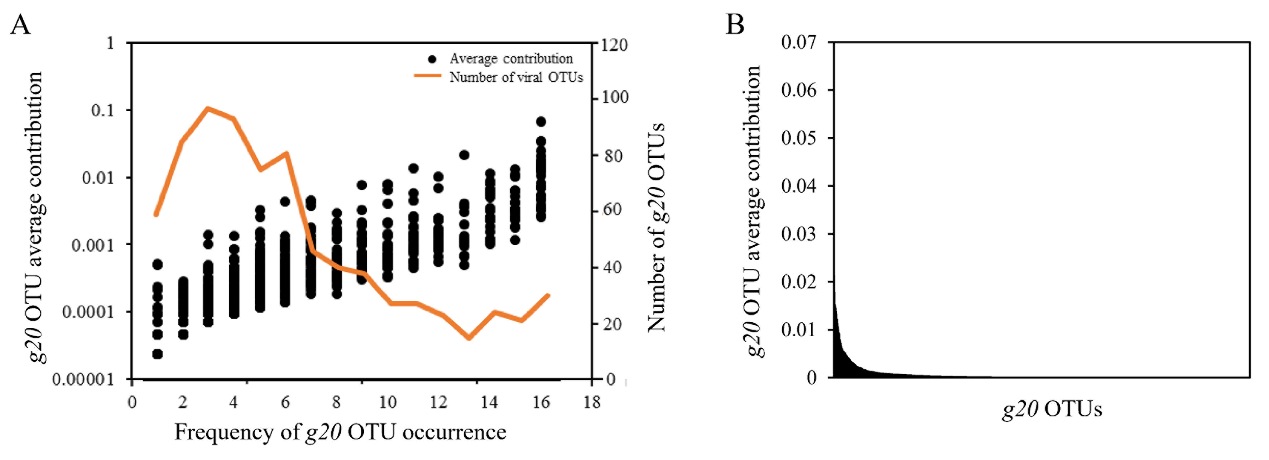


**Fig.** **S2** Frequency (A) and abundance rank (B) plots for *g20* OTUs. A spline curve and scatter diagram were used to express the relationship between the frequency of *g20* OTU occurrence and the number of *g20* OTUs as well as the average contribution of *g20* OTUs to the community, respectively. Each black dot represents an individual *g20* OTU.


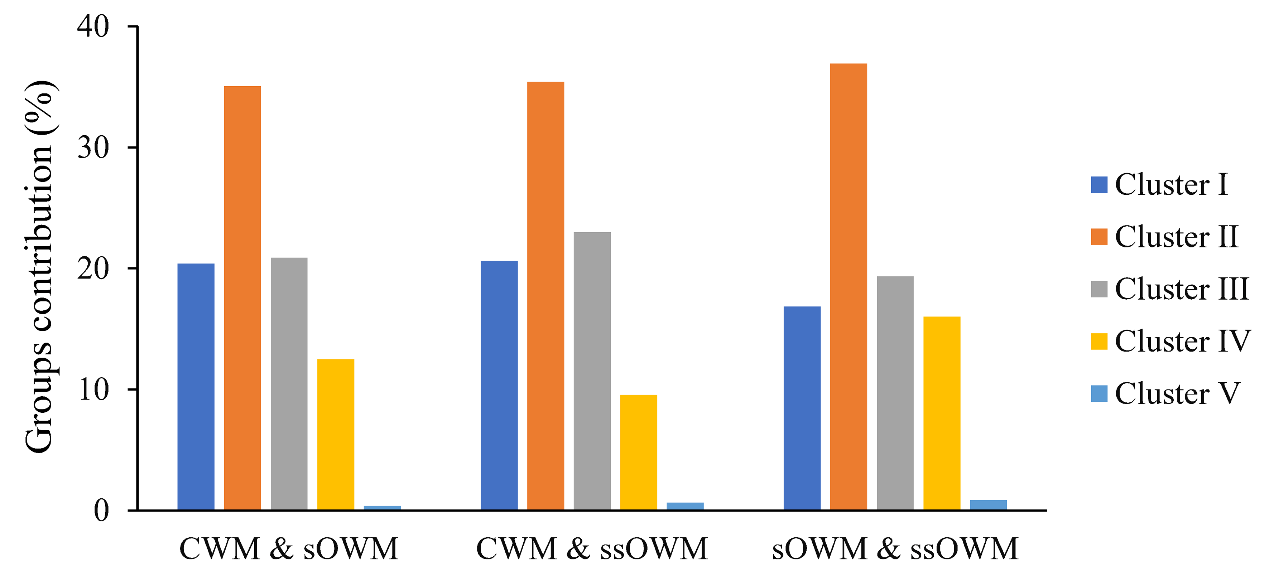
**Fig. S3** SIMPER analysis of the dissimilarity among four *g20* cyanophage oceanic water masses (cut off of low contribution = 90%).


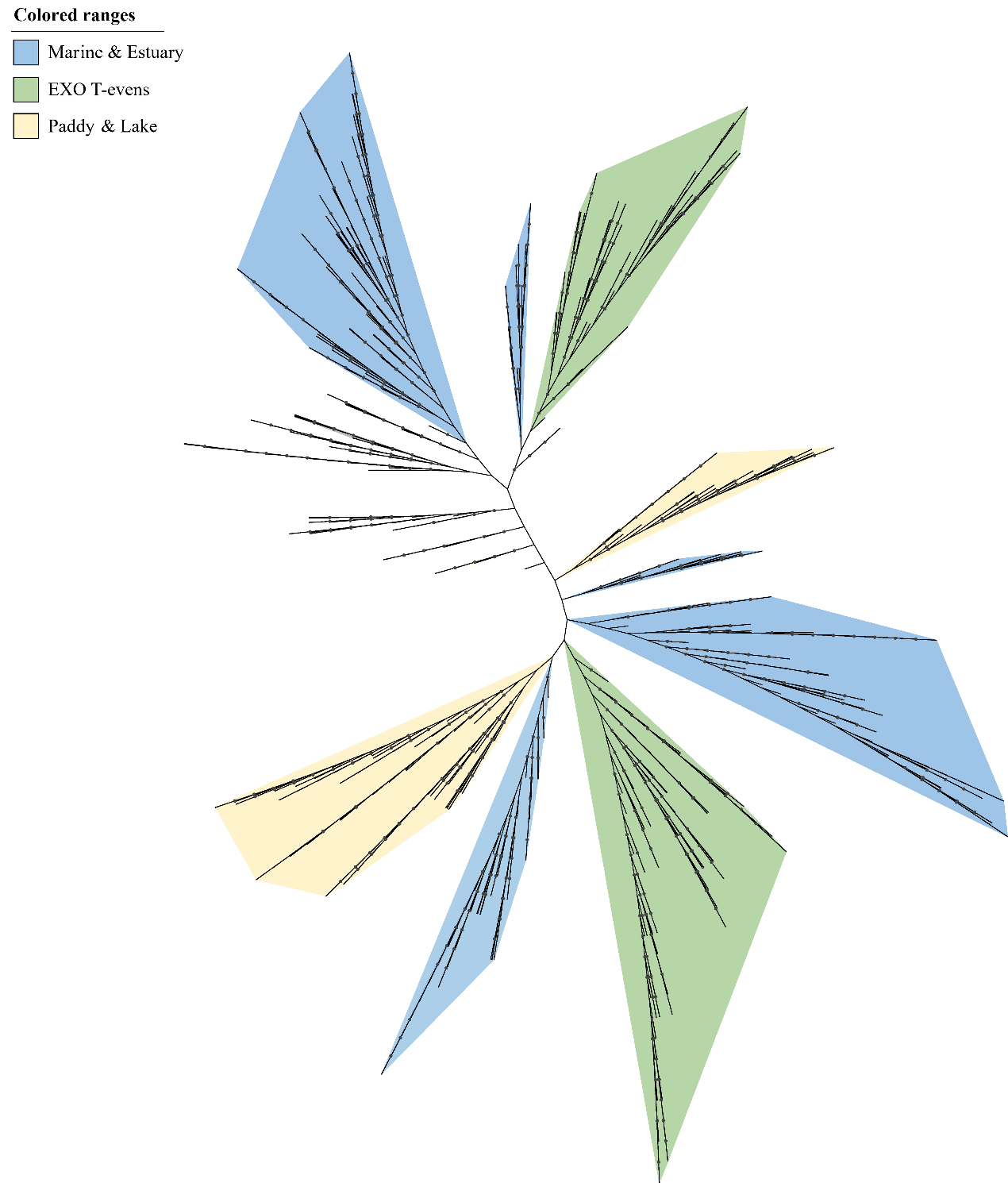


**Fig. S4** Maximum-likelihood phylogenetic analysis based on amino acid sequences of the obtained *g23* OTUs. Black dots show internal nodes with >90% bootstrap.


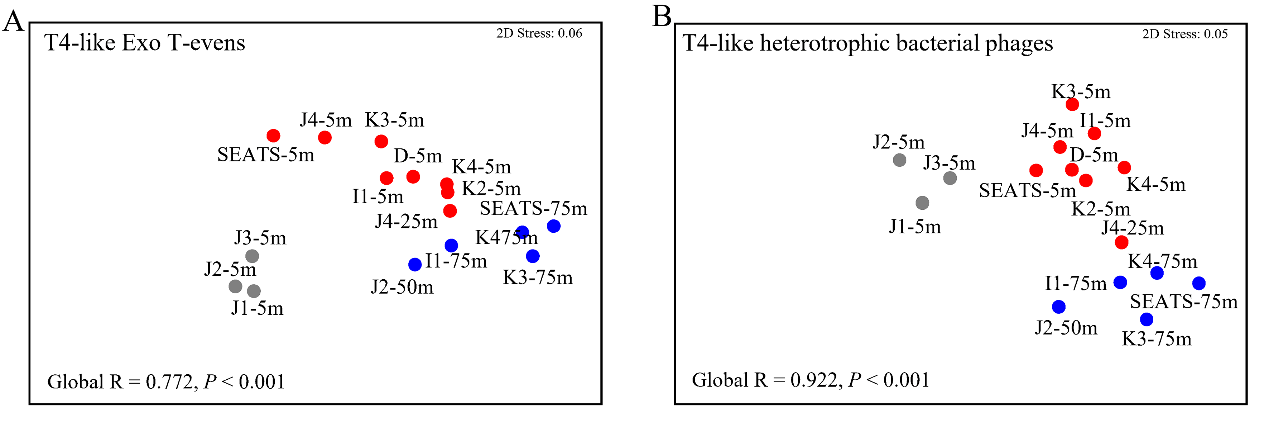


**Fig. S5** NMDS of the T4-like Exo T-evens (A), and T4-like heterotrophic bacteriophages (B) community based on all *g23* genes. Different oceanic water masses are marked in grey (CWM), red (sOWM), and blue (ssOWM), respectively.


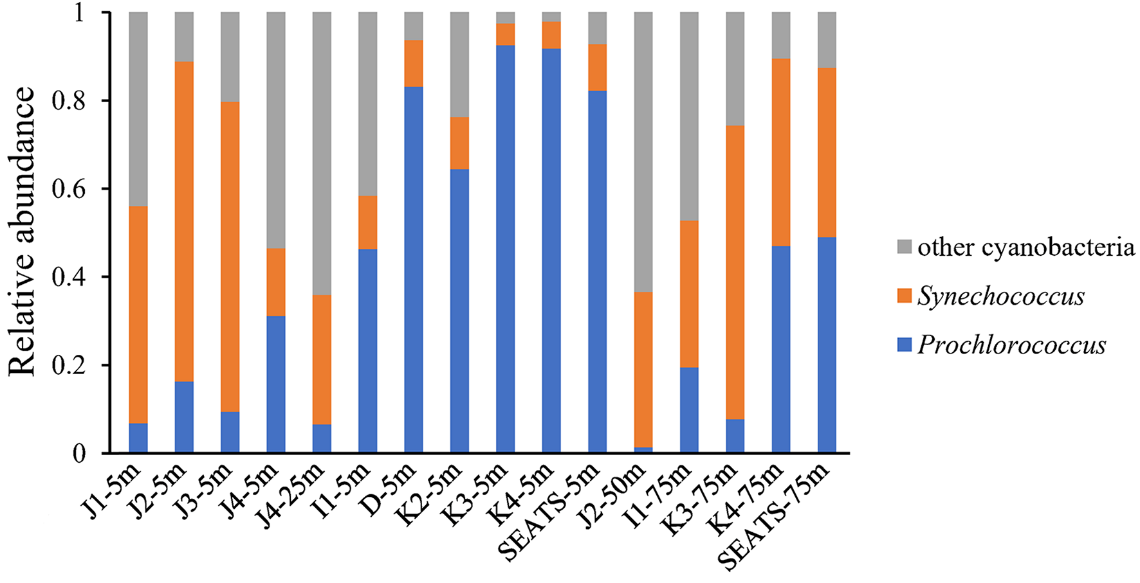


**Fig. S6** Cyanobacterial community biodiversity and composition

**Table S1** The Good’s coverage, richness (Chao I), diversity (Shannon and Simpson), and evenness indices across all *g20* samples at a 97% similarity level.

| Sample | Chao I | Coverage (%) | Shannon | Simpson | Evenness |
| --- | --- | --- | --- | --- | --- |
| J1-5m | 563 | 94.52 | 4.718 | 0.9799 | 0.3191 |
| J2-5m | 432 | 95.61 | 4.470 | 0.9752 | 0.2844 |
| J3-5m | 507 | 94.49 | 4.718 | 0.9812 | 0.3246 |
| J4-5m | 644 | 94.75 | 4.734 | 0.9770 | 0.2908 |
| J4-25m | 547 | 93.17 | 4.687 | 0.9796 | 0.3065 |
| I1-5m | 2105 | 94.37 | 5.891 | 0.9937 | 0.4258 |
| D-5m | 671 | 79.74 | 4.871 | 0.9805 | 0.3127 |
| K2-5m | 833 | 92.91 | 5.350 | 0.9879 | 0.392 |
| K3-5m | 545 | 92.54 | 4.757 | 0.9791 | 0.3206 |
| K4-5m | 707. | 91.22 | 4.829 | 0.9752 | 0.2731 |
| SEATS-5m | 704 | 94.49 | 5.254 | 0.9885 | 0.4106 |
| J2-50m | 550 | 96.10 | 4.345 | 0.9605 | 0.2328 |
| I1-75m | 716 | 92.12 | 5.235 | 0.9879 | 0.3856 |
| K3-75m | 453 | 95.50 | 4.850 | 0.9823 | 0.3789 |
| K4-75m | 449 | 93.29 | 4.550 | 0.9702 | 0.2817 |
| SEATS-75m | 550 | 94.56 | 4.667 | 0.9740 | 0.2980 |

**Table S2** Community comparison of *g20* cyanophage, T4-like Exo T-evens, and T4-like heterotrophic bacteriophages based on ANOSIM analysis with 999 permutations. **P* < 0.05, ***P* < 0.01 and ****P* < 0.001.

|  |  | CWM | sOWM | ssOWM |
| --- | --- | --- | --- | --- |
| *g20* cyanophage | CWM | - | - | - |
|  | sOWM | 0.949^**^ | - | - |
|  | ssOWM | 0.877^*^ | 0.809^**^ | - |
| T4-like Exo T-evens | CWM | - | - | - |
|  | sOWM | 0.93* | - | - |
|  | ssOWM | 1* | 0.458** |  |
| T4-like heterotrophic bacteriophages | CWM | - | - | - |
|  | sOWM | 0.960** | - | - |
|  | ssOWM | 1* | 0.830*** | - |
